# The Training of Health Visitors

**Published:** 1921-11

**Authors:** Joseph Cates

**Affiliations:** (County Medical Officer of Health, Surrey)


### THE TRAINING OF HEALTH VISITORS.

By JOSEPH CATES, M.D., D.P.H. (County Medical Officer of Health, Surrey).

Ten years ago there seemed little hope of unanimity of opinion among medical officers of health concerning the qualifications of health visitors. Some administrators in the public-health service considered that general nursing training was necessary, some thought a certificate of the Central Midwives Board sufficient, while others advanced the view that it was essential that candidates for appointment should be able to ride a cycle.

During the last decade many changes have occurred in the outlook of the sanitarian. Health visiting has passed beyond the experimental stage, and now has become a definite career for women, providing a field of employment the boundaries of which were daily increasing, until the present wave of reckless economy furnished a fashionable cry at the hustings.

The range and the type of the work has undergone considerable alteration; the duties of a health visitor now include most of the public health activities which can best be allotted to a woman—for example, maternity and child welfare, school nursing, the visiting of tuberculosis, measles, whooping-cough, and other infectious diseases, the inspection of midwives, of out-workers, and premises where women are employed. The salaries of the service, although still inadequate to attract enough well-trained women, are generally much higher than they were in 1911. The number of local authorities now appointing superintendent health visitors is increasing, and as these positions are reasonably well paid, they provide another goal for the ambitious.

In the early days of health visiting a great deal of pioneer work was done by voluntary workers, some of whom became salaried officers. Then midwives were appointed in certain areas, while in other districts fever nursing or some short period of hospital training was considered desirable. Soon, however, fully trained nurses began to take up health visiting, with the result that now the majority of health visitors are trained nurses, and in many localities the staff of health visitors is composed entirely of them.

The tide of opinion is setting strongly in favour of full nursing training for health visitors. At the Congress of the Royal Sanitary Institute held at Newcastle in 1920 there was much opposition to the suggestion that such training was unnecessary and even undesirable for women to be engaged in public-health work. A year later, at the Folkestone Congress, a paper expressing the conviction that a four years' course of hospital work was advisable met with no adverse criticism.

Advocates of a full course of hospital training urge that nursing is a career for women comparable to the medical profession for men, and that just as a medical officer of health has first to be a medical man, and later a specialist in public health, so a health visitor should pass through a recognised training-school for nurses and afterwards obtain additional qualifications in hygiene. The value of hospital life to the future health visitor is well expressed in the words of the reader of a paper at a recent discussion *:

> During the time she has spent in hospital she will have acquired a great deal of knowledge which will be indispensable to her in public-health work. An experience of discipline, self-control, self-reliance, precision, observation, tact, and loyalty, extending over some three years. In ward management she learns to be controlled and to control others, to consider the fact that she is one of a community and must exert herself for the common good.
>
> These qualities are essential to a woman who would undertake even a small position of authority, and cannot possibly be assimilated by a candidate for the health visitors' course prescribed by the Board of Education, a course made up of a few months spent here and a few months spent there, studying varying subjects in the hope that, combined, they may make a good whole.
>
> Practical experience in the nursing of acute illness, attendance in out-patient departments, in accident wards, and in special departments set apart for certain diseases and defects of the eye, ear, nose, and throat, are the best foundations on which to build up a knowledge of the prevention of disease. Every day we hear that it is wrong to separate treatment and prevention in the education of medical men. Do not the same reasons hold good in the training of a health visitor?

The chief objections of the opponents of nursing training are that the hospital emphasises too much the treatment and the cure of disease, and pays too little attention to the teaching of anatomy, physiology and hygiene, and, further, that ward discipline is unsuitable for one who will later visit the homes of the poor. It is admitted that the theoretical side of nursing has been somewhat neglected in the past, but great changes are taking place in the training-schools under the stimulating influence of the General Nursing Council and the College of Nursing, and it is almost certain that within a few months some of the largest institutions will provide a course which will not be open to the charge of insufficiency.

With regard to the second objection, a complete refutation is afforded in the successful work of the large number of trained nurses now engaged in health visiting.

The reader of the opening paper, in the discussion referred to above, outlined a course of training which has much to commend it. It was suggested that hospital training should begin early—at eighteen or nineteen years of age—and continue for four years. During this period opportunities should be given for attendance at lectures and for practical experience in public-health work, so that within the four years a nurse could obtain the certificate of the Central Midwives Board and the certificate of the Royal Sanitary Institute for Health Visitors and School Nurses. At the end of the training provision should

* Miss H. Weir, Superintendent Health Visitor, Northumberland County. "The Trained Nurse in Health Work." R.S.I. Congress, 1921.

**Training of Health Visitors—(*cont.*).**

be made by local authorities for the appointment of probationer health visitors at a living wage.

The problem to be faced in considering the training of health visitors is not so much needs of to-day or to-morrow, but the possibilities and demands which will face administrators engaged in public-health work ten years hence. Nor must one neglect to view the position from the side of the prospective health visitor; nursing is a profession of which a woman may be proud—and self-supporting. The opponents of the trained nurse as health visitor should pause before they help to institute a hybrid career which will please nobody but its sponsors, and will equip a woman neither for the public-health service nor for the nursing profession.

**Public Health Lectures for Nurses.**

The course of lectures which the College of Nursing has arranged in conjunction with the course in Public Health Nursing for International Students of the League of Red Cross Societies is full of interest not only to public-health nurses, but to all post-graduate nurses. Dr. Porter, Medical Officer of Health of Marylebone, Colonel Harrison, D.S.O., M.B., of St. Thomas's Hospital (who will speak on "Venereal Diseases"), and Miss Peterkin, Superintendent of Queen Victoria's Jubilee Institute for Nurses, are amongst the lecturers. Whilst practical subjects hold the field, the history of nursing will not be forgotten. Miss Coode, head of the preliminary training-school of St. Thomas's Hospital, will speak on this subject at the first meeting of the session, which is being held as we go to press. Another interesting subject chosen is on present-day methods of nursing old diseases. The lectures will be held each Tuesday, at 5.30 P.M., at St. Thomas's Hospital, and the fee is 5s. for the course, which lasts till April 4, with an interval of five weeks from December 6 to January 10. The full syllabus can be obtained from the Secretary to the College of Nursing, 7 Henrietta Street, Cavendish Square, W. 1. The provincial Local Centres of the College are also actively promoting courses of lectures for their members during the coming winter.

Writing of Health Week propaganda, "National Health" pays a compliment to medical officers of health and their work, as follows :—

We make no apology for considering the Medical Officer of Health to be the potential central force for this propaganda effort, albeit he may be, and usually is, a very much occupied official with whom we have the greatest sympathy. He has the basic information at command. He knows the perils of false economy. He can view with scientific eye the past, the present, and the future. He, enthusiastic about his work and its development, is embarrassed by the present financial crisis of the nation, and realises how greatly necessary a strong public opinion in favour of maternity and child welfare and general public health must be. To him, therefore, we look for guidance in Health Week propaganda. He obviously is the man of the moment.

